# A Constant Pressure-Driven Podocyte-on-Chip Model for Studying Hypertension-Induced Podocytopathy Pathomechanism and Drug Screening

**DOI:** 10.3390/mi16101097

**Published:** 2025-09-27

**Authors:** Yun-Jie Hao, Bo-Yi Yao, Qian-Ling Wang, Zong-Min Liu, Hao-Han Yu, Yi-Ching Ko, Hsiang-Hao Hsu, Fan-Gang Tseng

**Affiliations:** 1 Department of Engineering and System Science, National Tsing Hua University, Hsinchu 30013, Taiwan; hyjtb2009@gmail.com (Y.-J.H.); poi89420@gmail.com (B.-Y.Y.); wbetty01401@gmail.com (Q.-L.W.); miles151212@gmail.com (Z.-M.L.); yuhaohan92@gmail.com (H.-H.Y.); 2 Department of Nephrology, Kidney Research Center, Chang Gung Memorial Hospital, College of Medicine, Chang Gung University, Taoyuan 33305, Taiwan; koyc0429@gmail.com; 3 Institute of Nano Engineering and Microsystems, National Tsing Hua University, Hsinchu 30013, Taiwan; 4 Frontier Research Center on Fundamental and Applied Sciences of Matters, Hsinchu 30013, Taiwan; 5 Department of Chemistry, National Tsing Hua University, Hsinchu 30013, Taiwan; 6 Research Center for Applied Sciences, Academia Sinica, Taipei 11529, Taiwan

**Keywords:** podocyte, hypertension, recovery, organ-on-chip model

## Abstract

Podocytopathy, characterized by proteinuria, contributes significantly to kidney diseases, with hypertension playing a key role in damaging podocytes and the glomerular filtration barrier (GFB). The lack of functional in vitro models, however, impedes research and treatment development for hypertensive podocytopathy. We established a novel constant pressure-driven podocyte-on-chip model, utilizing our previously developed dynamic staining self-assembly cell array chip (SACA chip) and 3D printing. This platform features a differentiated podocyte monolayer under controlled hydrostatic pressures, mimicking the epithelial side of the GFB. Using this platform, we investigated mechanical force-dependent permeability to three sizes of fluorescent dextran under varying hydrostatic pressures, comparing the results with a puromycin aminonucleoside (PAN)-induced injury model. We observed that external pressures induced size-dependent permeability changes and altered cell morphology. Higher pressures led to greater macromolecule infiltration, especially for larger dextran (70 kDa, 500 kDa). Mature podocytes exhibited immediate, pressure-dependent cytoskeleton rearrangements, with better recovery at lower pressures (20 mmHg) but irreversible injury at higher pressures (40, 60 mmHg). These morphological changes were also corroborated by dynamic mRNA expression of cytoskeleton-associated proteins, Synaptopodin and ACTN4. This platform offers a promising in vitro tool for investigating the pathomechanisms of hypertension-induced podocytopathy, performing on-chip studies of the GFB, and conducting potential drug screening.

## 1. Introduction

Renal abnormality has become common and prevalent worldwide, due to the evolving challenges of modern lifestyles, an accelerating pace of life, the global aging population, and significant disparities in access to healthcare, facilities, and treatment among regions and countries [1]. According to studies from the International Society of Nephrology (ISN), over one tenth of the world population is suffering from kidney diseases, including acute kidney injury (AKI), chronic kidney disease (CKD), and kidney replacement therapy (KRT) [2]. Among these, CKD impacts the widest range of people and poses the greatest challenges to both human health and global economies, particularly given its heterogeneous origin, limited therapy, and the demands of long-term management [3]. Different from the reversible and preventable AKI, symptoms at the early stage of CKD are usually less evident [4]. Limited awareness among most individuals persists until they are diagnosed in irreversible late stages, leaving them with no viable options other than dialysis or transplantation. Among the various risk factors for chronic renal dysfunction, including obesity, heart disease, smoking, infections, family history or inherited conditions, lack of water or exercise, and chronic diseases like diabetes and hypertension, the latter are particularly highlighted and remain the most common leading causes, increasingly threatening millions globally, especially the elderly [5].

Damage or dysfunction of the kidney is usually grouped into five stages by referencing the guidance of the Kidney Disease Outcomes Quality Initiative (KDOQI), in which proteinuria and glomerular filtration barrier (GFB) impairment are typically involved at all stages [6]. From investigations of histopathologic lesions, many renal diseases are associated with glomerular dysfunction and a common determinant for various glomerular diseases including about 90% of nephrotic syndromes, is the dysfunction, injury, or loss of podocytes, especially in some clinical cases such as minimal change disease (MCD), diabetic kidney disease (DKD), membranous nephropathy, and focal segmental glomerulosclerosis (FSGS) [7].

Podocytes are terminally differentiated epithelial cells within the glomeruli and glomeruli are specialized capillary networks with capillary walls possessing a trilaminar structure—the fenestrated endothelium, glomerular basement membrane (GBM), and visceral epithelium. The fenestrated endothelium is formed by the glomerular endothelial cells (GECs), the GBM is produced by GECs and podocytes, and the visceral epithelium is developed by the terminally differentiated podocytes themselves. This unique structure contributes to ultrafiltration by the GFB, selectively filtering blood plasma from vessels to generate the primary urine collected by the surrounding Bowman’s capsule [8]. This dynamic process can only be maintained through the sustained integrity and functionality of the GFB, although mesangial cells are another essential component, providing structural support and being involved in regulating blood flow and matrix turnover [9]. Damage to these components will result in pathological changes and the failure of this special micro-physiological interface, whose function is determined by the transcapillary membrane pressure—also known as the effective filtration pressure (EFP)—the net force of the hydrostatic pressures and osmotic pressures between the blood and capsule, a crucial factor for maintaining its function [10]. This net filtration force is primarily driven by the intraglomerular hydrostatic pressure, which pushes fluid out of the glomerular capillaries, opposed by the capsular hydrostatic pressure and blood colloid osmotic pressure [11]. The intraglomerular hydrostatic pressure within the glomeruli is not measurable non-invasively [12]. Elevated intraglomerular pressure (intraglomerular hypertension) has been confirmed as the primary tensile stress that induces greater tensions in glomerular capillary walls, which subsequently challenges the mechanical maintenance of the internal homeostasis within glomeruli, often overwhelming the compensatory mechanisms involving the elasticity and contractility of their components [13].

Mature podocytes characterized by unique molecular regulations regarding cell cycle, mitosis, and cytoskeleton arrangement, have a limited ability to proliferate and self-renew as their progenitors [13]. Moreover, podocytes are highly sensitive and responsive to various mechanical forces and biochemical cues, undergoing a series of dysfunctional changes such as hypertrophy, detachment, reorganization of their actin-based cytoskeleton, and potential apoptosis [14,15]. In vivo studies indicate that while a podocyte loss of less than 20% can trigger compensatory hypertrophy to preserve function, irreversible glomerulosclerosis usually develops after more than 20% loss of podocytes [16].

Many engineered platforms have been developed to simulate the sophisticated glomeruli in vitro, including those providing stem cell-derived spheroids within co-culture niches to explore developmental potential [17], generating biomaterial curvature to recapitulate in vivo glomerular structure [18], being directly fabricated on chips with parallel channels separated by a functionalized porous membrane or GBM components, often integrated with perfusion systems (with [19,20,21] or without pumps [22]). Although both shear stress and hydrostatic pressure are important hemodynamic factors for simulating a functional GFB in vitro, most systems prioritize shear stress to mimic vascular flows from microvessels within the glomerulus, with only a few focusing on the impact of hydrostatic pressure differences on podocytes and their pressure-determined permeability [23,24].

To better study podocyte biology and pressure-induced podocytopathy, we developed a constant pressure-driven podocyte-on-chip model. This model builds up on the dynamic staining self-assembly cell array chip (SACA chip) [25] and a glomerular leakage model developed previously in our group [26]. The primary purpose of this platform is to facilitate the investigation of mechanical force-dependent podocyte permeability and the potential subsequent recovery of these cells following stimulation. We successfully cultured and differentiated a monolayer of podocytes on the bottom side of the chip to mimic the epithelial side of the GFB. Supported by 3D-printed scaffolds, the chip was maintained in a closed system with controlled constant pressures provided by an external micropump. We then examined changes in the permeability of monolayered podocytes in response to different hydrostatic pressures at immediate and later time points. These results were compared with those from an in vitro disease model resulting from puromycin aminonucleoside (PAN) treatment. PAN is a known podocytotoxin, commonly used to induce podocyte nephrosis for studying podocyte injury with proteinuria. For our experiments, three different hydrostatic pressures were selected to simulate low, medium, and high intraglomerular hydrostatic pressure conditions in vivo [27], respectively. Beyond permeability, we also observed actin-associated morphological changes and relevant molecular evidence regarding the sensitive response of podocytes. Our functional platform enables the direct in vitro investigation of the pathomechanism behind intraglomerular hypertension-induced podocytopathy. Furthermore, its modular design allows it to be expanded to facilitate real-time imaging of injury responses at the single-cell level, long-term interactive crosstalk studies between podocytes and other glomerular cells, early biomarker screening for podocytopathy, and the exploration of podocyte recovery following mechanical stimulation to prevent proteinuria.

## 2. Materials and Methods

### 2.1. Establishment of the Microfluidic Chip and Chip-Based Platform

**Figure 1:**
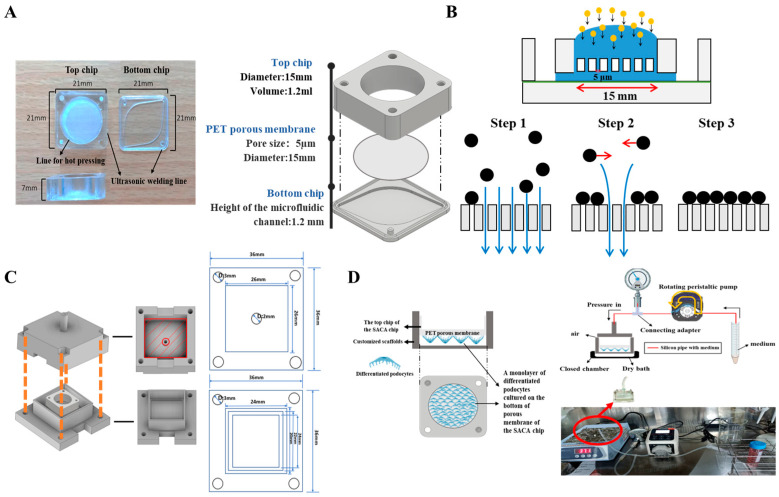
Schematic images to show the cell-separation dynamic staining self-assembly cell array chip (CSDS-SACA chip), the mechanism, and the chip-customized scaffolds for controlling the external pressure in this study. (**A**) Three-dimensional images of the CSDS-SACA chip and its parameters; (**B**) Illustration of the mechanism to form a monolayer of cells by the CSDS-SACA chip; (**C**) Illustrations of the design of the chip-customized scaffolds for external pressure control. The upper and lower parts of each scaffold feature four holes at their corners, which are used to fasten the parts together using screws and nuts. The upper part includes a hollow central extrusion for connecting to a pressure meter, which allows for precise control of the internal scaffold pressure after assembly. (**D**) Schematic images of the set of organ-on-chip model, based on the CSDS-SACA chip, established in this study and the real image of the entire platform used in this study. Sketches of scaffolds were made by using Inventor pro 2022 (Autodesk® inventor professional 2022, Autodesk Inc., San Francisco, CA, USA) and some icons in figures were from BioRender (BioRender.com).

The chip used in this study is a component of the cell-separation dynamic staining self-assembly cell array chip (CSDS-SACA chip) previously developed in our group. Its design is based on our prior work on the dynamic staining self-assembled cell array (SACA) chip [25] and the three-dimensional microwell dialysis (3D-μDialysis) chip [28]. The top chip features a translucent, microporous, hydrophilic, track-etched polyester (PET) membrane film (5 µm pore size, 4 × 10^5^ pores/cm^2^ pore density) (Sterlitech, Sterlitech Corporation, Auburn, WA, USA) that was cut and bounded to a membrane holder. This holder was composed of cyclic block copolymers (CBCs) (ViviOnTM) and fabricated via injection molding. The bottom chip, also produced from CBCs by injection molding, contains a streamlined channel for flow distribution. The configuration of the entire chip is displayed in Figure 1A. As an improvement over the 3D-μDialysis chip, the 15mm diameter top chip is designed to form a monolayer of up to 15–20 million cells on its membrane (step 3). This monolayer formation is driven by a vertical flow distribution resulting from the porous membrane (step 1), and horizontal shear stress derived during cell sedimentation (step 2) (Figure 1B). Simulations detailing the flow distribution during cell sedimentation inside the top chip are provided in the Supplementary Materials.

In this chip-based platform, different types of cells can be cultured on both sides of the porous membrane of the top chip. In our current model, a monolayer of mouse podocytes was cultured and differentiated on the bottom side of the membrane of the top chip. The top side of the membrane can be used for co-culturing endothelial cells, intraglomerular mesangial cells, immune cells, and stem cells, which allows for future studies on direct cell–cell interactions and facilitates the mimicking of the complete glomerular filtration barrier (GFB) microenvironment. To apply external pressure, scaffolds customized for the chip were 3D-printed based on the dimensions of the chip (Figure 1C). A set of scaffolds, silicon pipelines, a peristaltic pump, and a pressure meter were then assembled to form a pressure-controlled platform for external hydrostatic pressure stimulation. To prevent leakage, a cushion pad made of polydimethylsiloxane (PDMS) or rubber was placed on the inner surface of the 3D-printed scaffold. The two parts of the scaffold were then fastened together with screws and nuts inserted through corresponding holes at the four corners of the assembly (Figure 1C). Schematic images of the chip-based organ-on-chip platform with controlled pressure are displayed in Figure 1D.

### 2.2. Cell Culture

Mouse podocytes with stabilized expressions of green fluorescent protein (mpodocyte-GFP) were kindly provided by Dr. Hsu and cultured/differentiated in vitro as previously described [26]. In brief, this conditionally immortalized podocyte cell line, featuring a temperature-sensitive T-antigen was maintained in a proliferation state at 33. The growth medium consists of Roswell Park Memorial Institute (RPMI) 1640 (10-040-CM, Corning, NY, USA) 10% fetal bovine serum (FBS) (Corning, NY, USA), 100 U/mL of penicillin and 100 mg/mL of streptomycin (Penicillin Streptomycin Solution, 100×, Corning, NY, USA), 10 IU/mL of mouse interferon-gamma (mouse IFN-g research grade, Miltenyi Biotec B.V. & Co. KG, Bergisch Gladbach, Germany), and 250 µg/mL of geneticin (G418, Gibco, Life Technologies Ltd., Paisley, UK). To inactivate the T-antigen and induce podocyte differentiation, the growth medium was replaced with a differentiation medium containing RPMI1640, 10% FBS, and 1% Penicillin Streptomycin Solution. Cells were then differentiated at 37 °C for at least 12 days prior to use in experiments [26].

Before seeding podocytes on the PET membrane of the chip, type I collagen solution (3 mg/mL, porcine collagen type I, Expercy Medical Ltd., Taipei City, Taiwan) was pre-coated. To examine the ability of podocytes to proliferate and differentiate on the chip, a series of cell numbers were first tested to identify appropriate seeding density for both plate and chip cultures (Supplementary Materials). Cell proliferation was examined by measuring the cell viability using the CCK-8 assay on days 1, 3, 5, and 7 post-seeding. Subsequently, to compare cell proliferation between 24-well plates and pre-coated chips, an identical number of cells was seeded in both formats, and cell viability was measured on days 1, 3, 5, and 7 post-seeding using the CCK-8 assay.

### 2.3. CCK-8 Assay

Podocytes cultured in a T25 flask at 33 °C were trypsinized (0.25% Trypsin solution, 15-053-CI, Corning, NY, USA) and counted when they reached 80–90% confluence. After seeding them in 24-well microplates and collagen type I-coated chips, cells were placed back into the incubator (33 °C and 5% CO_2_). The viability of cells cultured in plate and chips were then examined at different days post-seeding, using the CCK-8 assay, following instructions of the manufacturer. Briefly, CCK-8 buffer (Elabscience^®®^, Elabscience Biotechnology Co., Ltd., Wuhan, China) was added to wells and chips containing podocytes at each time point. After 4 h incubation (at 33 °C for proliferation assessment) in dark, the optical density (OD) of the supernatant of each sample were measured using a microplate reader (BioTeck Epoch 2 Microplate Spectrophotometer, Agilent, Santa Clara, CA, USA). Data were calculated and plotted using Microsoft Excel (Microsoft Office, Redmond, WA, USA). Each experimental group consisted of at least three replicates (n = 3), and every test was repeated three times (N = 3).

### 2.4. External Pressure and Puromycin Aminonucleoside Treatment

A monolayer of mpodocyte-GFP cells was cultured and differentiated on the chip for at least 12 days as previously described in Section 2.2.

To prepare for pressurization, the cell-loaded chip was placed in the bottom scaffold. Differentiation medium containing fluorescent dextran was added to the top of the membrane on the chip, while differentiation medium without fluorescent dextran was added to the bottom scaffold. Following this, the cell-loaded chip was carefully placed into the bottom scaffold. To ensure a closed environment, the top scaffold was positioned over the bottom one and locked using screws and nuts (Figure 1C). The assembled scaffold was then connected to a pressure meter and a peristaltic pump via silicon pipes. External pressure was controlled by running the pump, with the pressure monitored in real-time on the pressure meter. Once the desired external pressure (20, 40, or 60 mmhg) was reached, the pump was stopped. To maintain stable pressure and prevent leakage, a section of silicon pipe was filled with a certain volume of sterilized PBS, acting as a buffer to prevent fluid from entering the scaffold chamber. The pressurization was performed in a laminar flow hood to maintain a sterile and biocompatible condition. The pressurized scaffold was kept in a dry bath device at 37 °C in the dark for 3 h.

To prepare for PAN treatment, the half-maximal inhibitory concentration (IC50) of the puromycin aminonucleoside (PAN) against differentiated podocytes was first determined using the CCK-8 assay (Supplementary Materials). To do this, cells were cultured and differentiated in 96-well microplates. A series of PAN concentrations, ranging from 2.4 to 200 µg/mL (including 2.4, 4.8, 9.6, 19.2, 25, 50, 100, 150, and 200 µg/mL) were administrated to the cells in three replicate wells per concentration. Following a 72 h administration period, cell viability was assessed via CCK-8 assay and the IC50 was determined by fitting a dose–response curve to the resulting OD values. Based on this 72 h IC50, two concentrations of PAN (20 and 100 µg/mL) were then selected to induce varying degrees of podocyte injury on our organ-on-chip platform, for comparison with the effects of external pressure. Two cell-loaded chips were used for a PAN treatment group, with a corresponding control group treated with a normal differentiation medium.

### 2.5. Immunofluorescent Staining

To confirm the differentiation of podocytes and their morphology, specific markers were visualized using immunofluorescent staining, including Wilms’ tumor protein (WT-1), Zonula occludens-1 (ZO-1), and Podocin. Briefly, samples were fixed by 2% polyformaldehyde solution for approximately 10 min at room temperature. This was followed by rinsing with Dulbecco’s Phosphate-Buffered Saline (DPBS, Corning, USA), and permeabilization with 0.5% Trito X-100 for 10 min. Following another DPBS rinse, non-specific interactions were blocked by incubating samples with 1% Bovine Serum Albumin (BSA, A9647, Sigma, St. Louis, MO, USA) solution (dissolved in DPBS) overnight at 4 °C. Subsequently, primary antibodies, diluted in 1% BSA, were applied for overnight incubation at 4 °C. These included antibodies against WT-1 (1:100, Wilms Tumor 1 Recombinant Mouse Monoclonal Antibody (rWT1, 857), NeoBiotechnologies, Union City, CA, USA), ZO-1 (1:100, Monoclonal Antibody (ZO1-1A12), Invitrogen, Waltham, MA, USA), and Podocin (1:100, Podocin Polyclonal Antibody, Bioss, Beijing, China). Following three DPBS rinses, fluorescent-conjugated secondary antibodies (1:1000, ab6939 Goat Anti-Rabbit IgG H&L (Cy3^®®^), ab97035 Goat Anti-Mouse IgG H&L (Cy3^®®^) pre-adsorbed, or ab97115 Donkey Anti-Goat IgG H&L (Cy3^®®^) pre-adsorbed) were applied for 1 h incubation in the dark at room temperature. After another three DPBS rinses, samples were ready to be visualized under a fluorescent microscope (Olympus IX71, Tokyo, Japan). To assess cytoskeleton and cell morphology after treatments, filamentous actin (F-actin) was visualized using a high-affinity F-actin probe conjugated with a photostable green fluorescent Alexa Fluor 488 dye (ActinGreen 488 ReadyProbes, Invitrogen, Waltham, MA, USA) and DAPI (Invitrogen, Waltham, MA, USA) was employed to counterstain nuclei.

### 2.6. Permeability of Fluorescent Macromolecules Measurement

To assess the permeability of the platform in response to external pressures and chemical drugs, fluorescent dextrans with three different molecular weights and fluorophores (DX20-FC-1, 20 kD; DX70-S5-1, 70 kD; and DX500-RB-1, 500 kD; Nanocs Inc., New York, NY, USA) were applied. The specific protocol varied depending on the stimulus.

For assessing the pressure-induced permeability, the three fluorescent dextrans were added to the medium before hydrostatic pressure stimulation. Following a 3 h administration period of pressures, medium was collected from the lower chamber of the platform, and its fluorescent intensity was measured using a plate reader (GloMax^®®^ Explorer Multimode Microplate Reader, Promega, Madison, WI, USA).

For assessing permeability following PAN treatment, differentiation medium with fluorescent dextran was added to the top of the membrane, while differentiation medium without dextran was added to the bottom chamber. After a three-hour incubation, medium was collected from the bottom of the membrane and its fluorescent intensity was measured using a plate reader (GloMax^®®^ Explorer Multimode Microplate Reader, Promega, Madison, WI, USA).

To normalize the permeability results, the fluorescent intensity of differentiation medium without three sizes of fluorescent dextran was also measured to serve as a background control. For comparison, samples without pressure or drug treatment were run in parallel as the experimental control group.

### 2.7. RNA Isolation and qPCR Analysis

To evaluate the mRNA expression of specific markers in differentiated podocytes cultured in our platform after treatments (e.g., different pressures or PAN concentrations), total RNA was extracted from treated and untreated samples using the AllPrep DNA/RNA/Protein Mini Kit (Qiagen, Germantown, MD, USA), according to the instructions from the manufacturer. Subsequently, relative mRNA expressions of specific protein markers Synaptopodin (Mm_Synpo_2_SG QuantiTect Primer Assay) and alpha-Actinin 4 (Mm_Actn4_1_SG QuantiTect Primer Assay) were quantified on the Mic qPCR Cycler (Bio Molecular Systems, Coomera, Australia) by two-step qRT-PCR (QuantiTect^®®^ SYBR^®®^ Green PCR Kit, Qiagen, Germantown, MD, USA), following the procedure outlined by the manufacturer. Glyceraldehyde 3-phosphate dehydrogenase (GAPDH) (Mm_Gapdh_3_SG QuantiTect Primer Assay) was used as an inner control for normalization. The concentrations of total RNA and cDNA were measured using the take 3 plate on a microplate reader (BioTeck Epoch 2 Microplate Spectrophotometer, Agilent, Santa Clara, CA, USA).

### 2.8. Statistical Analysis

For all assays, data were presented as means ± standard deviation. The optical density (OD) values from the CCK-8 assay and the normalized fluorescent intensity of permeated molecules were first processed in Microsoft Excel (Microsoft Office, USA), and then statistically analyzed in Origin^®®^ pro 2017 (OriginLab, Northampton, MA, USA). Permeability data were normalized relative to the blank control, defined as the fluorescent intensity of differentiation medium without fluorescent molecules. Changes in cell morphology were monitored and quantified using fluorescent images and the ImageJ software (ImagJ 1.54f Fuji) [29]. To quantify, three images per treatment or control group were acquired with a CMOS camera connected to an Olympus IX71 fluorescent microscope. From each image, data from 50 randomly selected cells were collected using the ROI manager in ImageJ (Supplementary Materials), to measure cell area and aspect ratio.

One-way ANOVA with Tukey’s post hoc test was performed to compare differences between groups. All significance levels were defined as follows and are indicated with symbols on the plotted figures. Between experimental groups and the control group, if the *p* value is less than 0.001, then this significance of the group will be labeled as ***; if *p* value is less than 0.01, then the significance will be labeled as **; if *p* value is less than 0.05, then the significance is labeled as *. Between a recovering group and its corresponding experimental group, if the *p* value is less than 0.001, then this significance of the group will be labeled as ◆◆◆; if the *p* value is less than 0.01, then this significance of the group will be labeled as ◆◆; if the *p* value is less than 0.05, then this significance of the group will be labeled as ◆.

For qRT-PCR results, GAPDH was used as the endogenous control for all samples. The relative mRNA expression level of specific markers was quantified using the 2^−∆∆CT^ method [28]. Data were then analyzed using an ordinary one-way ANOVA followed by Dunnett’s multiple comparisons test and plotted with GraphPad Prism (version 8.0.0 for Windows, GraphPad Software, San Diego, CA, USA, www.graphpad.com). The statistical significance in figures was determined by following *p*-value thresholds, if the *p* value for the experimental group compared with the control group was less than 0.001, the significance was labeled as ***; if the *p* value was less than 0.01, the significance was labeled as **; if the *p* value was less than 0.05, the significance was labeled as *.

## 3. Results

### 3.1. Establishement of the Podocyte-on-Chip Platform

**Figure 2:**
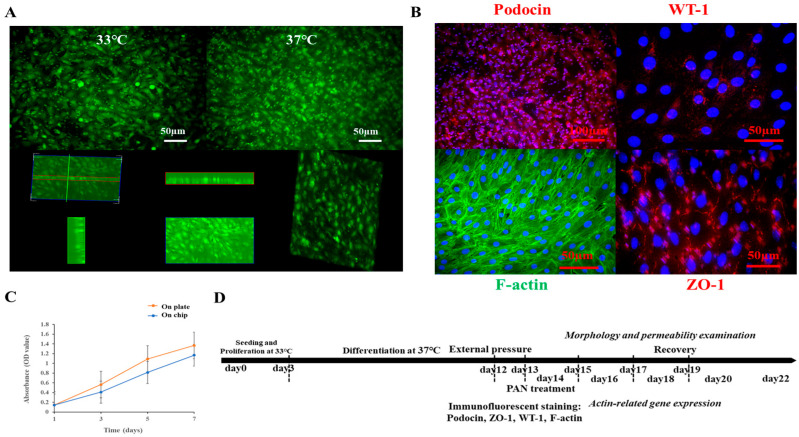
Illustrations of the proliferation and differentiation of mouse podocytes cultured on the chip. (**A**) Fluorescent images to show the undifferentiated and differentiated mouse podocytes with GFP expression cultured on the chip at 33 °C and 37 °C, respectively, and the monolayer of differentiated podocytes on the chip. (**B**) Immunofluorescent images to show specific markers expressed by differentiated mouse podocytes cultured on the chip-based platform; specific markers including podocin, WT-1, and ZO-1 were visualized by secondary antibody with cy3 conjugation. Cytoskeleton F-actin was labeled by phalloidin-FITC, and nuclei were counterstained by the DAPI. Scale bars on fluorescent images were 50 or 100 µm. (**C**) Comparison of the proliferation of mouse podocytes cultured on the chip and normal tissue culture plate; the proliferation of cells was examined by CCK-8 essay. (**D**) Time axis to display the establishment of the podocyte-on-chip model, including cell proliferation, cell differentiation, stimulations by external pressure or drug, and function assessments after treatments.

To establish the organ-on-chip platform, the ability of mouse podocytes with a stable GFP expression to proliferate and differentiate on the coated top chip was first compared with their behavior on a standard 24-well tissue culture plate (Figure 2 and Supplementary Materials). Proliferation was assessed using the CCK-8 assay, while differentiation was visualized by immunofluorescent staining. Our results showed that seeding podocytes at an appropriate density yielded comparable proliferation rates on both the coated chip and the tissue culture plate (Figure 2C). Based on these findings, the organ-on-chip platform was established. Podocytes were first seeded on the chip and proliferated for two days at 33 °C. Subsequently, they were differentiated for at least 12 days at 37 °C before functional assays (Figure 2D). To monitor both proliferation and differentiation, cell morphology was regularly observed (e.g., at day 1, 3, 5, 7, 9, 12) and documented via a fluorescent microscope (Olympus IX71). Fluorescent images captured during culture demonstrated the formation of a confluent podocyte monolayer and revealed key morphological differences between the proliferation and differentiation stages (Figure 2A). To confirm successful differentiation, the expression of specific markers, including the podocin, ZO-1, WT-1, and the cytoskeleton marker F-actin, was visualized by immunofluorescent staining (Figure 2B).

### 3.2. Effect of Different External Pressures on Podocyte Morphology on the Platform

**Figure 3:**
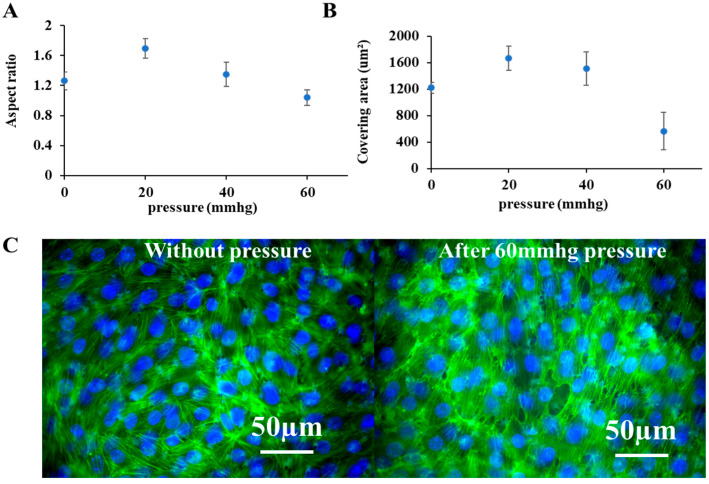
Morphological changes in cells in response to external pressures. (**A**,**B**) Changes in the aspect ratio and covering area of living mouse podocytes with GFP expression cultured on the platform, based on the monitoring of fluorescent images of cells before and after 3 h external pressure treatment (Supplementary Figures); To quantify the aspect ratio and covering area of cells, each sample was taken three images randomly at different places under a fluorescent microscope and 50 individual cells were selected manually on every image using ImageJ (Supplementary Figures). (**C**) Immunofluorescent images of the cytoskeleton of mouse podocytes cultured on the platform with and without external pressure. Cytoskeleton F-actin was labeled by phalloidin-FITC, and nuclei were counterstained by the DAPI. Scale bars in merged images were 50 µm.

To monitor and quantify morphological changes in cells in response to external pressure and during recovery, fluorescent images of the cells were captured with a fluorescent microscope before, during, and after stimulation. The aspect ratio (Figure 3A) and covering area (Figure 3B) of individual cells were quantified from these images to reflect morphological reorganization. From the results, following pressure application, cells rounded, and their area decreased, reflecting significant morphological reorganization compared with unpressurized controls. These changes were further visualized and validated with immunofluorescent staining of the cytoskeleton (Figure 3C). Unlike the smooth, intact cytoskeleton of control cells, the pressurized cells exhibited a rough margin and disrupted cell–cell connections, directly reflecting the induced injury. Representative fluorescent images of living cell morphologies are provided in the Supplementary Materials.

### 3.3. Effect of Different External Pressures on Podocyte Permeability on the Platform

**Figure 4:**
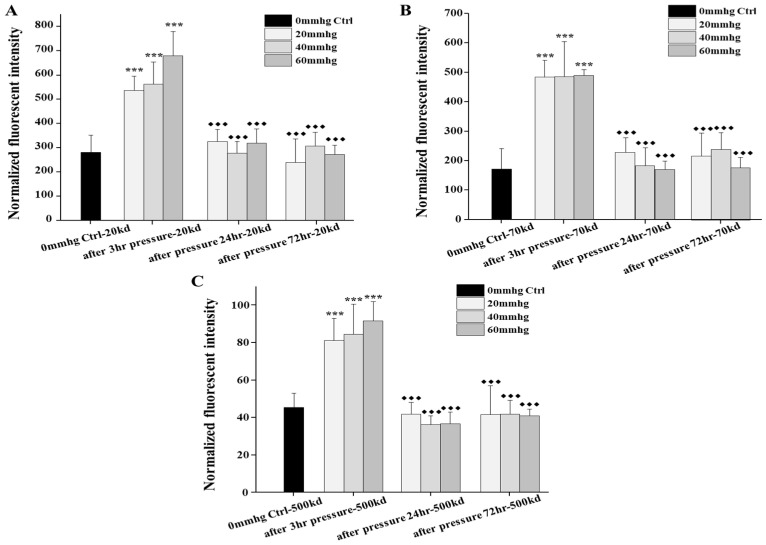
Comparisons of permeabilities of fluorescent dextran by our organ-on-chip platform in response to different external pressures immediately and during recovery in the following 72 h after treatment. (**A**–**C**) Comparisons of the permeabilities of the 20 kd, 70 kd, and 500 kd fluorescent dextran by our platform after 3 h different external pressures immediately and during recovery in the following 24 and 72 h after treatment, with no pressure control, respectively. For the pressure-induced permeability, each test was examined on two chips at the same time and repeated at least three times (n = 2, N = 3–4). No pressure control (n = 2, N = 3) was examined to monitor the external pressure test, and blank control was examined to normalize results of fluorescent dextran by plate reader. To compare results, one-way ANOVA with Tukey’s post hoc test was analyzed between chips with and without pressure. The star symbol * denoted the significance of pressured samples compared with no pressure control (if the *p* value is less than 0.001, then this significance of the group will be labeled as ***) and the black rhombus ◆ denoted the significance of recovering samples compared with pressured samples (if the *p* value is less than 0.001, then this significance of the group will be labeled as ◆◆◆).

The filtration function of the podocyte-on-chip platform was confirmed by examining the effect of external pressure on the permeability of fluorescent dextrans with three different sizes (20, 70, and 500 kD). As external pressure increased, there was a significant increase in infiltrated fluorescent dextran of all three sizes in the lower chamber medium. This was observed immediately after pressurization, with significant differences between pressurized and unpressurized control chips (Figure 4).

The platform also demonstrated size-selective filtration, as smaller dextran molecules were more easily infiltrated than larger ones. It was observed that in one specific experiment, the permeability to 20 and 70 kD dextran increased even at lower pressures (20 and 40 mmHg), while the infiltration of the 500 kD molecules significantly increased only after high-pressure treatment (60 mmHg) (Supplementary Materials).

The potential recovery ability of podocytes after stimulation was also preliminarily investigated by monitoring the permeability at 1 and 3 days following pressure application. A significant difference in permeability was observed between the immediately pressurized chips and those measured 24 and 72 h later, suggesting the potential for the compensation and recovery of podocytes. Our preliminary results indicated that lower external pressures caused less damage and hence, resulted in better functional recovery. For example, in one specific experiment, it was observed that after 5 days of recovery, the platform blocked approximately 76% of all fluorescent molecules treated with 20 mmHg pressure, compared with only 22% after a 60 mmHg treatment (Supplementary Materials).

### 3.4. Effect of PAN on Podocyte Morphology and Permeability on the Platform

Puromycin aminonucleoside (PAN) was often applied to make proteinuric models due to podocyte injury in vitro. To compare the damage from external pressures on the function of our organ-on-chip platform, the effect of PAN on cell morphology and permeability of the organ-on-chip platform was also examined, serving as a positive control of podocyte injury model.

**Figure 5:**
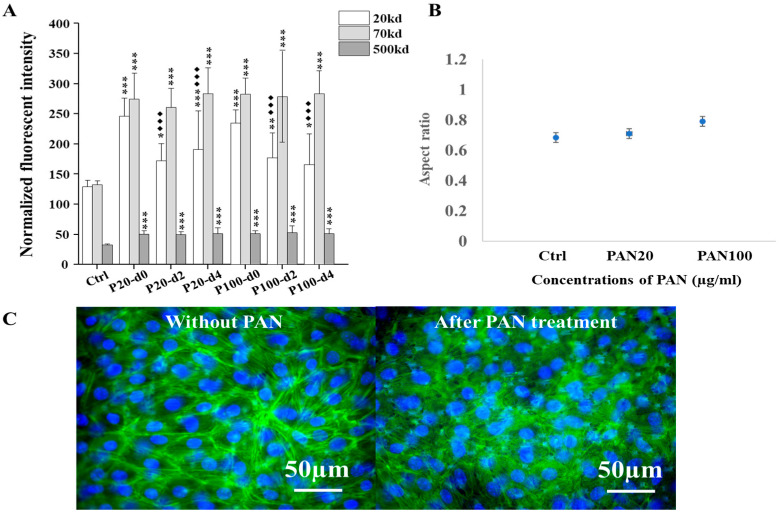
Comparisons of the permeabilities of fluorescent dextran and morphological changes in cells by our chip-based platform in response to PAN treatment in vitro. (**A**) Comparisons of the permeabilities of fluorescent dextran by our organ-on-chip platform after 20 and 100 µg/mL of PAN treatments for 3 days and during recovery in the following 4 days in vitro; For the PAN-induced permeability, each test was examined on two chips at the same time and repeated at least three times (n = 2, N = 3). Groups without PAN treatment (n = 2, N = 3) were examined as control. To compare results, one-way ANOVA with Tukey’s post hoc test was analyzed between chips with and without PAN treatment, and between recovering after treatment immediately and during the following days. The star symbol * denoted the significance of experimental groups compared with no PAN control group (if the *p* value is less than 0.001, then this significance of the group will be labeled as ***; if *p* value is less than 0.01, then the significance will be labeled as **; if *p* value is less than 0.05, then the significance is labeled as *), and the black rhombus ◆ denoted the significance of recovering groups compared with groups after corresponding treatment immediately (if the *p* value is less than 0.001, then this significance of the group will be labeled as ◆◆◆). (**B**) Changes in the aspect ratio of living mouse podocytes with GFP expression cultured on the platform, based on fluorescent images of cells with and without PAN treatment for 3 days (n = 2, N = 3). The IC50 of PAN was measured in tissue culture plate, the result of which is provided in Supplementary Materials. (**C**) Immunofluorescent images of the cytoskeleton of mouse podocytes cultured on the platform with and without PAN treatment. Cytoskeleton F-actin was labeled by phalloidin-FITC, and nuclei were counterstained by the DAPI. Scale bars on merged images were 50 µm.

The IC50 of PAN on differentiated podocyte was first determined (see Supplementary Materials). Based on these results, two concentrations of PAN (20 and 100 µg/mL) were selected to induce varying degrees of podocyte injury on the platform. After a 3-day treatment period, our results showed a significant increase in the permeability of fluorescent dextran of all three sizes (20, 70, and 500 kD) compared with the control. The number of dextran molecules permeating across the membrane significantly increased following PAN treatment (Figure 5A). Fluorescent image-based quantification also revealed morphological changes in cells after PAN treatment compared with control groups (Figure 5B). These changes were consistent with the permeability results. Furthermore, immunofluorescent staining showed a disrupted cytoskeleton and obvious disconnection between cells, confirming the cellular injury caused by PAN (Figure 5C).

To preliminarily assess the recovery, the permeability was also monitored for up to 4 days following PAN treatment. The damage to the podocytes was found to be largely irreversible. All experimental groups displayed significant differences in permeability compared with their corresponding untreated control group.

### 3.5. Effect of the External Pressure and PAN on the mRNA Expression of Synaptopodin and Alpha-Actinin 4

**Figure 6:**
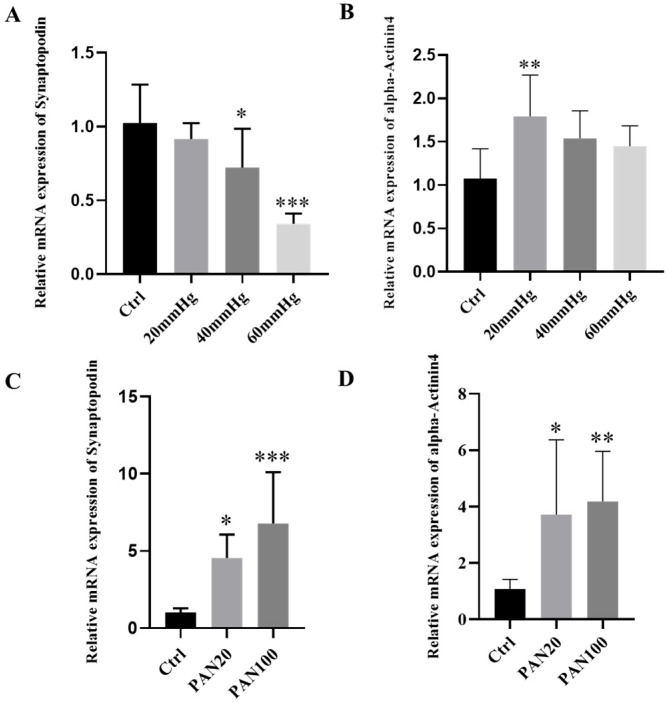
Relative mRNA expression of Synaptopodin and alpha-Actinin4 after treatments. (**A**,**B**) Relative mRNA expression of Synaptopodin and alpha-Actinin4 by podocytes on the organ-on-chip platform after different external pressure treatment (n = 3, N = 3); (**C**,**D**) Relative mRNA expression of Synaptopodin and alpha-Actinin4 by podocytes on the organ-on-chip platform after PAN treatment with different concentrations (n = 3, N = 3). Ordinary one-way ANOVA followed by Dunnett’s multiple comparisons test was examined. If the *p* value of the experimental group compared with the control group is less than 0.001, then this significance of the group in the plotted figure will be labeled as ***; if *p* value is less than 0.01, then the significance will be labeled as **; if *p* value is less than 0.05, then the significance is labeled as *.

Synaptopodin and alpha-Actinin 4 are both actin-filament-associated proteins, essential for maintaining the integrity of mature podocyte cytoskeleton. Our results displayed that the relative mRNA expression of both Synaptopodin and alpha-Actinin 4 was affected by external pressure in a pressure level-dependent manner (Figure 6A,B).

For effects of external pressure on the relative mRNA expression of Synaptopodin, it was significantly reduced in response to higher pressures (40, 60 mmHg), compared with the control group. For alpha-Actinin 4, in contrast, the relative mRNA expression presented significant differences only in the lower pressure-treated group (20 mmHg), compared with the control. It is noteworthy that while the relative expression was reduced, the overall transcript level of alpha-Actinin 4 was upregulated in podocytes following external mechanical stimulation

For effects of PAN treatment, similarly, the mRNA expression of both Synaptopodin and alpha-Actinin 4 was significantly affected by PAN. Our results demonstrated a PAN concentration-dependent upregulation of both Synaptopodin and alpha-Actinin 4 mRNA levels. The higher the concentration of PAN applied, the greater the expression of both genes, with a significant difference observed between all PAN-treated groups and the control group. The overall levels of expression for both markers were upregulated after PAN treatment (Figure 6C,D).

## 4. Discussion

In the glomerulus, differentiated podocytes with interdigitated foot processes form a special epithelial layer inside Bowman’s capsule. This layer, along with the glomerular basement membrane and the glomerular capillary endothelial layer, constitutes the glomerular filtration barrier (GFB). Its filtration function depends on the effective filtration pressure (EFP), which is the net balance of glomerular hydrostatic pressure, capsular hydrostatic pressure, and blood colloid osmotic pressure. Any disturbance to the physiological structure of this barrier or to the homeostatic balance of the mechanical forces that determine the EFP can impair its physiological function. As pressure-induced podocyte loss is a major factor in many glomerular diseases, numerous studies have demonstrated the inherent mechano-sensitivity of podocytes [30] and their adaptive responses to various mechanical forces [15], such as stretch, strain [15,31,32], tensile stress, and laminar fluid shear stress [33,34].

Based on a microfluidic chip previously developed by our group, we established a podocyte-on-chip platform supported by a set of 3D-printed scaffolds. We successfully simulated the variable permeability of the podocytes epithelial layer in response to dynamic transmembrane pressures in vitro. This system accurately mimicked glomerular leakage due to podocyte injury induced by elevated intraglomerular pressure, as different sizes of macromolecules infiltrated in a pressure-dependent manner. Moreover, the platform enabled us to explore the adaptive response and potential recovery of podocytes after mechanical stimulation. External pressures are typically mechano-transduced by activating mechanical sensors in the extracellular matrix (ECM) and plasma membrane (e.g., cell–cell/matrix contact proteins, ion channels) [35]. These sensors convert physical stimuli to biochemical signals, causing cellular adaptations such as the cytoskeleton reorganization and specific changes in related gene or protein expression [36,37]. In our study, following the administration of lower mechanical forces or lower concentrations of puromycin aminonucleoside for up to five days, the filtration barrier of mature podocytes on our platform displayed partial recovery. This was consistent with observed morphological changes such as alternations in cell aspect ratio and body size, which reflect the rearrangement of the cytoskeleton in response to external forces or the drug PAN. These cytoskeleton dynamics were further validated by immunofluorescent staining of the cytoskeleton component F-actin and the relative mRNA expression of actin-associated proteins (Synaptopodin and alpha-Actinin 4) in treated podocytes.

Synaptopodin is a key actin filament-associated protein specific to podocyte that influences the dynamics of the actin-based cytoskeleton. It serves as an important indicator of glomerular homeostasis and a biomarker for glomerular disease. Alpha-Actinin 4 (ACTN4), a member of the alpha-Actinin family, is widely expressed in non-muscle cells, where it helps organize, regulate, and stabilize the cytoskeleton. Beyond maintaining cell morphology and structure, this multifunctional protein also plays essential roles in cell adhesion, cell motility, cellular signal transduction, and nuclear transcription. Mutations or dysfunction in ACTN4 have been associated with kidney disorders like focal segmental glomerulosclerosis (FSGS), as well as metastatic cancers [38]. In our study, the decreased expression of Synaptopodin in podocytes on our platform in response to external pressure was consistent with results from other in vitro [26] and in vivo studies [39]. Similarly, the direct upregulation of alpha-Actinin 4 has also been reported in other drug-induced podocyte injury models, which often results in foot process effacement and proteinuria [40]. Our platform allowed us to observe variations in the expression of both Synaptopodin and alpha-Actinin 4 in response to different stimulations—indicating the reorganization of actin filaments within the cytoskeletons, a key adaptive response that facilitates morphological changes [41,42] to compensate for the disrupted physiological function induced by dynamic forces [43,44] or drugs [45,46]. Furthermore, our platform also provided insights into the synergistic relationship between Synaptopodin and alpha-Actinin 4 in maintaining cell morphology. Their distinct expression patterns in response to various stimuli correlated with the elongation and bundling of long actin filaments [47], as well as the upregulated alpha-Actinin 4 expression and the accumulation of actin-assembly proteins at cell–cell junctions [48]. These distinct expression patterns may be attributed to their different physiological functions and the signaling pathways that regulate the actin-mediated morphological architecture of differentiated podocytes in vivo.

For example, elevated intraglomerular pressure has been shown to directly induce mechanical stress on glomerular podocytes, leading to podocyte hypertrophy in vitro [32]. Within a certain threshold, this adaptive hypertrophy was not associated with damaged podocyte machinery, although the synthesis of certain structural components might be altered. It was the decompensated podocyte hypertrophy that ultimately led to podocyte loss and proteinuria, and might even contribute to focal segmental glomerulosclerosis [49]. It was also found that specific signaling pathways, such as the Wnt/β-catenin pathway, played important roles in the pathomechanism of podocyte injury and related proteinuria [50]. The adenosine 3′,5′-monophosphate (cAMP)-protein kinase A (PKA) pathway has also been implicated in regulating Synaptopodin gene expression and puromycin-induced morphological and functional changes in podocytes in vivo. While targeting these interconnected signaling networks may contribute to disease recovery [41], it has been proposed that the cyclooxygenase-2 (COX-2) PGE2-PGE2 receptor 2 (EP2)-involved signaling pathway, rather than the cAMP-PKA pathway, may mediate the mechano-transduction of the fluid flow shear stress and tensile stress towards structural and functional alternations in podocytes [51]. Recent studies have manifested that cytoskeleton rearrangement is highly correlated with podocytopathies, with numerous cytoskeleton components and signaling molecules, such as actin, microtubule intermediate filaments, calcium, transient receptor potential channels, myosin, and Rho family proteins, all playing a role [52]. The transiently formed sarcomere-like structure, in particular, is thought to be crucial for mechano-sensation, peripheral foot process formation and in vivo-like actomyosin contractility in podocytes [53].

The accumulating evidence suggests that different types of stimuli may activate distinct signaling pathways to induce specific regulatory cascades in vivo, although they can ultimately lead to similar pathological phenotypes. These complex interplays warrant further sophisticated study of the biomarkers and molecular pathways involved in podocytopathies, which holds promise for the design and development of potential drugs and medical interventions [54].

## 5. Conclusions

In conclusion, our newly developed functional podocyte-on-chip platform represents a promising avenue for creating a sophisticated in vitro glomerular model. While it provides valuable insights, it is important to acknowledge certain limitations, including its reliance on mouse podocytes, its simplified representation of the complex in vivo environment, the challenges in recapitulating long-term chronic conditions, and the basic design of the hydrostatic pressure-supplying apparatus. Nevertheless, this platform will be invaluable for investigating a range of conditions, such as the basal physiological mechanical microenvironment for podocyte maturation, hypertension-induced podocytopathy, and the progression of renal failure. It is also a robust system for studying mechanosensitive and mechano-transduction-related biomarkers and assessing potential interventions for recovering injured podocytes. Furthermore, it provides a powerful tool for screening drugs and therapeutic strategies aimed at maintaining podocyte homeostasis, thereby helping to prevent or alleviate proteinuria-associated podocytopathies and nephrotic syndromes. This work thus represents a significant step towards developing more predictive in vitro models for kidney disease research.

## Data Availability

The data supporting this article have been included in the main manuscript and as part of the Supplementary Materials. More information will be available on reasonable requests.

## Supplementary Materials

The following supporting information can be downloaded at https://www.mdpi.com/article/10.3390/mi16101097/s1, Figure S1: Proliferation curve and seeding density optimization of mouse podocytes. Figure S2: Representative images to display how to quantify the morphological changes in mouse podocytes cultured on our platform with and without treatment. Figure S3: Representative fluorescent images of living mpodocyte-GFP cultured on the platform before and after different pressurizations. Figure S4: Representative images of living mpodocyte-GFP cultured on the platform before and after treatments. Figure S5: One specific function and recovery test, and the IC 50 of PAN treatment (3 days) on mouse podocytes. Figure S6: The PET membrane of the SACA chip and the simulation of the flow distribution to drive cells into the monolayer by the chip.
