# Supplementary material for: A Constant Pressure-Driven Podocyte-on-Chip Model for Studying Hypertension-Induced Podocytopathy Pathomechanism and Drug Screening
Source: Micromachines (Basel). 2025 Sep 27;16(10):1097. doi: 10.3390/mi16101097 (PMC12566487; doi:10.3390/mi16101097)
Supplement: Supplementary file 1 [file micromachines-16-01097-s001.zip › micromachines-3751016-supplementary.pdf]

Supplementary data

A Constant Pressure-Driven Podocyte-on-Chip Model for Studying Hypertension-induced Podocytopathy Pathomechanisms and Drug Screening

Yun-Jie Hao,[a] Bo-Yi Yao,[a] Qian-Ling Wang,[a] Zong-Min Liu,[a] Hao-Han, Yu,[a] Yi-Ching Ko,[b] Hsiang-Hao Hsu, *[b] and Fan-Gang Tseng*[a c d e f]

[a.] *Department of Engineering and System Science, National Tsing Hua University, Hsinchu, Taiwan.*

[b.] *Department of Nephrology, Kidney Research Center, Chang Gung Memorial Hospital, Chang Gung University, College of Medicine, Taoyuan 33305, Taiwan.*

[c.] *Institute of Nano Engineering and Microsystems, National Tsing Hua University, Hsinchu 30013, Taiwan.*

[d.] *Frontier Research Center on Fundamental and Applied Sciences of Matters, Taiwan.*

[e.] *Department of Chemistry, National Tsing Hua University, Hsinchu, 30013, Taiwan*

[f.] *Research Center for Applied Sciences, Academia Sinica, Taiwan*

*Correspondence: hsianghao@gmail.com and fangang@ess.nthu.edu.tw (F.-G.T.); Tel.: +886-3-571-5131 (ext. 34270) (F.-G.T.)

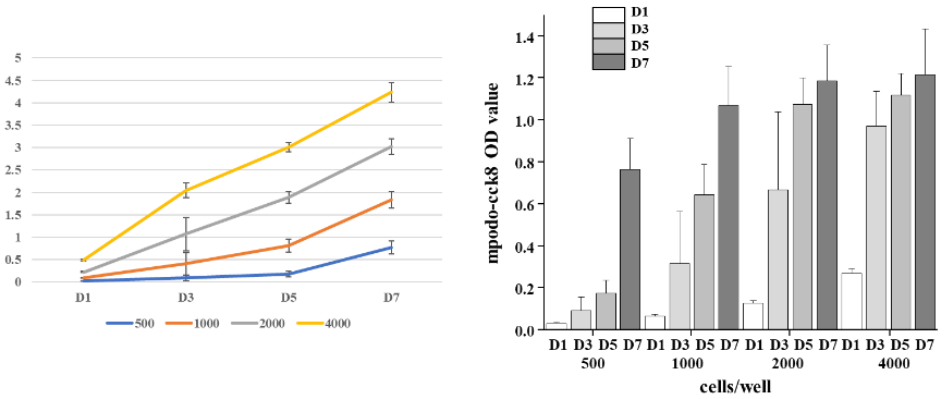

Supplementary figure S1: Proliferation curve and seeding density optimization of mouse podocytes. A series of cell numbers were first tested the proliferation curve to identify appropriate seeding density for both plate and chip cultures. Cell proliferation was examined by measuring the cell viability using the CCK-8 assay on days 1, 3, 5 and 7 post-seeding.

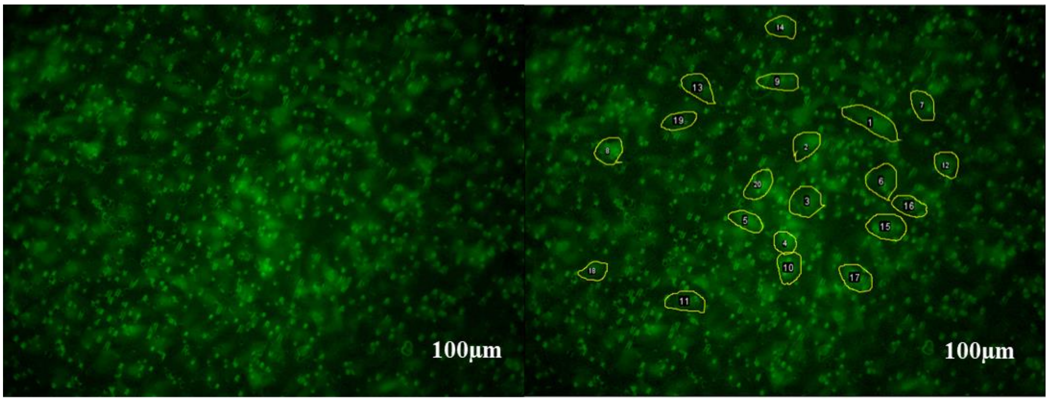

Supplementary figure S2: Representative images to display how to quantify the morphological

changes of mouse podocytes cultured on our platform with and without treatment. For calculating the aspect ratio and covering area, data from 50 individual cells were selected randomly using the ROI manager (a tool of Image J software) and quantified.

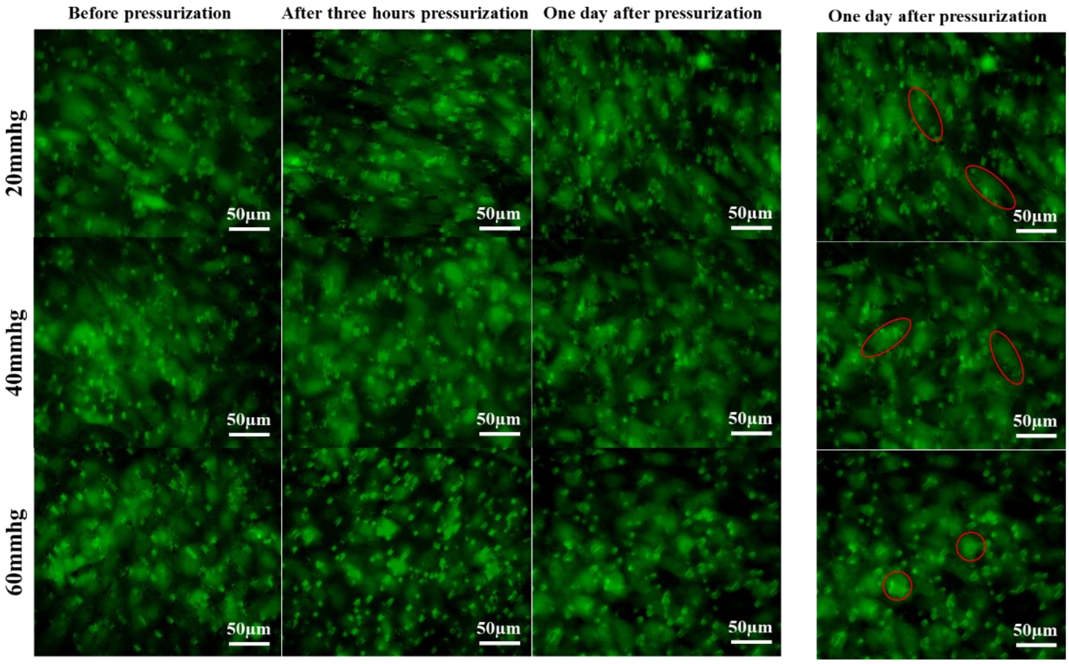

Supplementary figure S3: Representative fluorescent images of living mpodocyte-GFP cultured on the platform before and after different pressurizations.

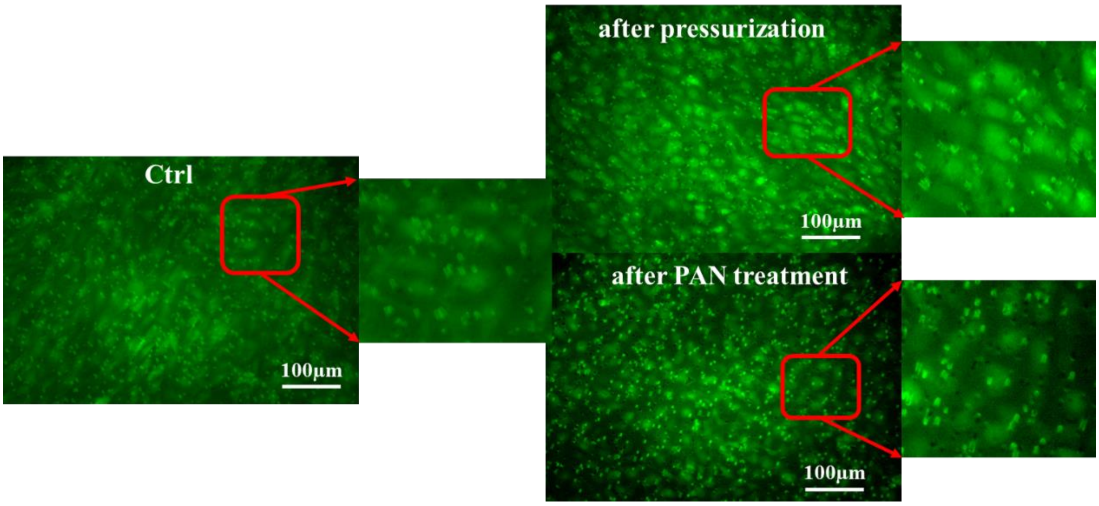

Supplementary figure S4: Representative fluorescent images of living mpodocyte-GFP cultured on the platform before and after different stimulations.

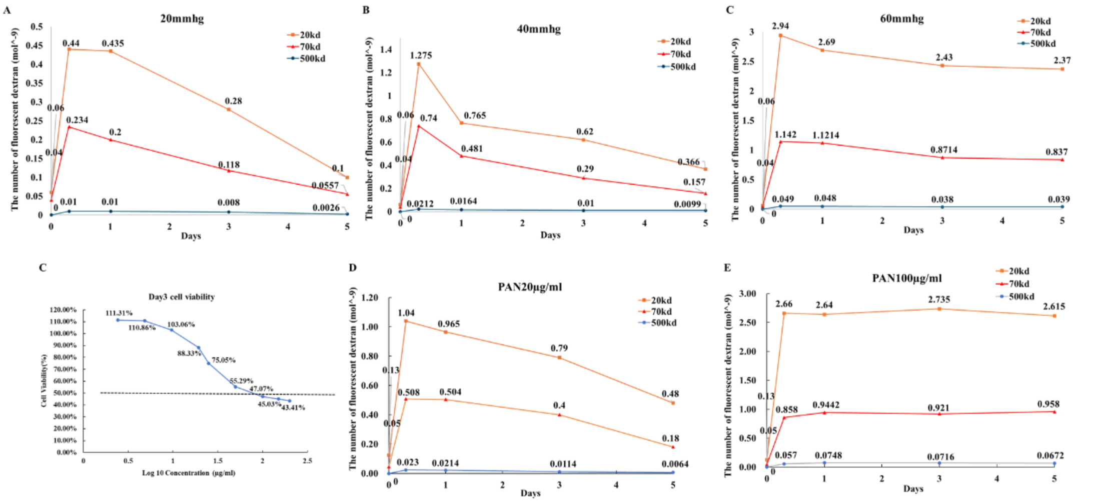

Supplementary figure S5: One specific function and recovery test, and the IC 50 of PAN treatment (3days) on mouse podocytes

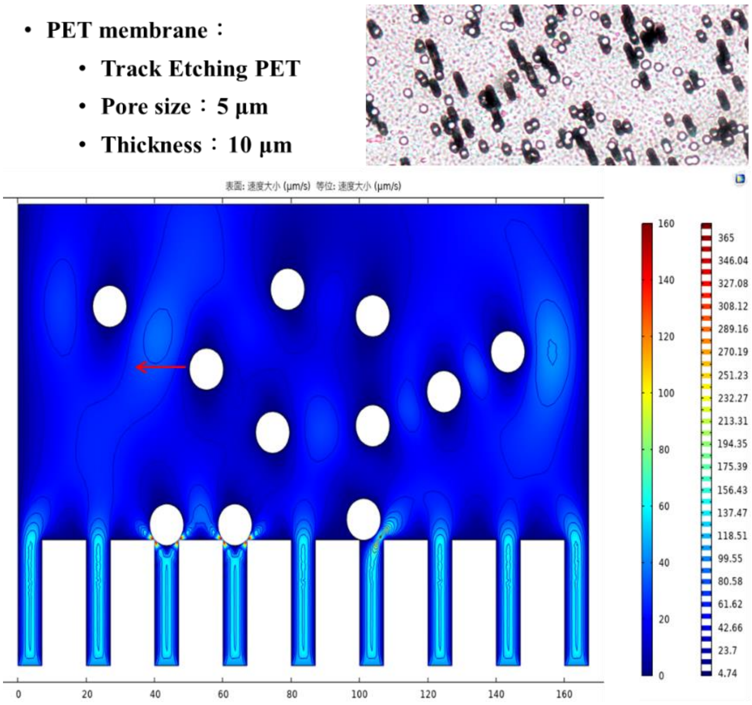

Supplementary figure S6: The PET membrane of the SACA chip and the simulation of the flow distribution to drive cells into monolayer by the chip.
